# Vesicular Axonal Transport is Modified In Vivo by Tau Deletion or Overexpression in *Drosophila*

**DOI:** 10.3390/ijms19030744

**Published:** 2018-03-06

**Authors:** Yasmina Talmat-Amar, Yoan Arribat, Marie-Laure Parmentier

**Affiliations:** Institut de Génomique Fonctionnelle (IGF), Université de Montpellier, Centre National de la Recherche Scientifique (CNRS), Institut National de la Santé et de la Recherche Médicale (INSERM), 34094 Montpellier, France; yasmina.talmat@sanofi.com (Y.T.-A.); yoan.arribat@unil.ch (Y.A.)

**Keywords:** microtubule, axonal transport, tauopathy, Alzheimer’s disease

## Abstract

Structural microtubule associated protein Tau is found in high amount in axons and is involved in several neurodegenerative diseases. Although many studies have highlighted the toxicity of an excess of Tau in neurons, the in vivo understanding of the endogenous role of Tau in axon morphology and physiology is poor. Indeed, knock-out mice display no strong cytoskeleton or axonal transport phenotype, probably because of some important functional redundancy with other microtubule-associated proteins (MAPs). Here, we took advantage of the model organism *Drosophila*, which genome contains only one homologue of the Tau/MAP2/MAP4 family to decipher (endogenous) Tau functions. We found that Tau depletion leads to a decrease in microtubule number and microtubule density within axons, while Tau excess leads to the opposite phenotypes. Analysis of vesicular transport in *tau* mutants showed altered mobility of vesicles, but no change in the total amount of putatively mobile vesicles, whereas both aspects were affected when Tau was overexpressed. In conclusion, we show that loss of Tau in *tau* mutants not only leads to a decrease in axonal microtubule density, but also impairs axonal vesicular transport, albeit to a lesser extent compared to the effects of an excess of Tau.

## 1. Introduction

Axons are specific cellular structures extending along large distances in adult organisms. Their integrity requires a robust cytoskeleton, which also enables anterograde and retrograde active transport between the soma and the synaptic terminals. Microtubules (MTs) are the tracks required for axonal transport and their organization and stability are controlled by different structural microtubule-associated proteins (MAPs). The MAP2/Tau family contains three genes in Vertebrates encoding the proteins MAP2, MAP4, and Tau [1]. These proteins contain a similar microtubule-binding domain, responsible for microtubule stabilization and bundling [2,3,4,5]. Numerous in vitro studies or studies based on overexpression of these proteins indicated that they are able to promote MT polymerization, MT stabilization, and MT bundling [6,7,8,9,10].

Tau is also known to be the major component of the intracellular filamentous deposits (neurofibrillary tangles) that define a number of neurodegenerative diseases, called tauopathies. Primary tauopathies like frontotemporal dementia and Parkinsonism linked to chromosome 17 (FTDP-17) are due to mutations in the *tau* gene [11,12,13,14]. Secondary tauopathies, like Alzheimer’s disease (AD), display filamentous Tau deposits, but are not directly caused by mutations in Tau. In these diseases, neurofibrillary tangles contain hyper-phosphorylated forms of Tau that weakly bind to microtubules [15,16,17,18]. There is still a debate about the relative importance of toxic gain-of-function of hyper-phosphorylated pathological Tau and loss of normal Tau function in the disease progression, especially in the early phases of the disease [19,20,21]. Indeed, hyper-phosphorylated Tau multimers may be responsible for a toxic gain of function, by sequestering other proteins required for normal cell function [17]. On the other hand, Tau loss of function, especially its detachment from microtubules through phosphorylation, would lead to microtubule destabilization, defects in axonal transport and, in the long term, synaptic dysfunction and neuronal degeneration [22,23]. Because of this context, it is important to understand the function of endogenous Tau, especially considering microtubules and axonal transport.

However, the analysis of the consequences of Tau depletion, in Tau knock-out mice or in cell culture, revealed neither any major brain defects, nor any clear disruption of the MT cytoskeleton, nor any axonal transport defects [24,25,26,27]. This is in contrast with studies showing that an excess of Tau impairs axonal transport [28,29,30,31]. This would suggest that loss of Tau has milder consequences than an excess of this protein. However, the mild phenotypes of Tau knock-out mice could also be the consequence of functional redundancy within the Tau/MAP2 family or with other MAPs.

Because of their lower genetic redundancy, invertebrate organisms are useful to assess the endogenous role of proteins like the MAP2/Tau proteins. In *Drosophila melanogaster*, Drosophila Tau is the only member of the MAP2/Tau family [1,32,33]. Hence, the analysis of microtubule structure and axonal transport in conditions of loss of Tau in this organism is of importance. The first study describing *tau* mutants in *Drosophila* did not reveal any decrease in the amount of MTs in oocytes [34]. A more recent study detected a change in the organisation of MTs, with decreased MT density in axons of adult brains [35]. However, up to now, no direct functional consequence of this structural defect on axonal transport has been described [35,36,37]. Here, we studied the effects of Tau depletion on vesicular axonal transport in *Drosophila* larval segmental nerves, which are particularly suitable to study axonal transport, and compared these effects with those obtained in conditions of an excess of *Drosophila* Tau. We first analysed and compared the effects of loss or overexpression of Tau on MT density in the axons present in these nerves. We found that loss of Tau leads to a decrease in axonal MT density while an excess of Tau leads to an increase in MT density. These changes in MT density were also accompanied by changes in axon calibre. Analysis of vesicular transport in *tau* mutants showed an increase in the pausing time of vesicles, but no change in the total amount of putatively mobile vesicles. In the presence of an excess of Tau, we also found an increase in pausing time of vesicles, but also a significant decrease in the total amount of putatively mobile vesicles.

In conclusion, our results show that decreased microtubule density in *tau* mutants is associated with defects in vesicular transport. However, the defects observed in axonal transport in the absence of Tau are milder compared to the defects observed with an excess of Tau. This indicates that an excess of Tau protein is much more detrimental for the neuronal physiology than a decrease in the amount of this protein, even in the absence of genetic redundancy between Tau, MAP2, and MAP4.

## 2. Results

### 2.1. Tau Is Present in Larval Segmental Nerves

**Figure 1:**
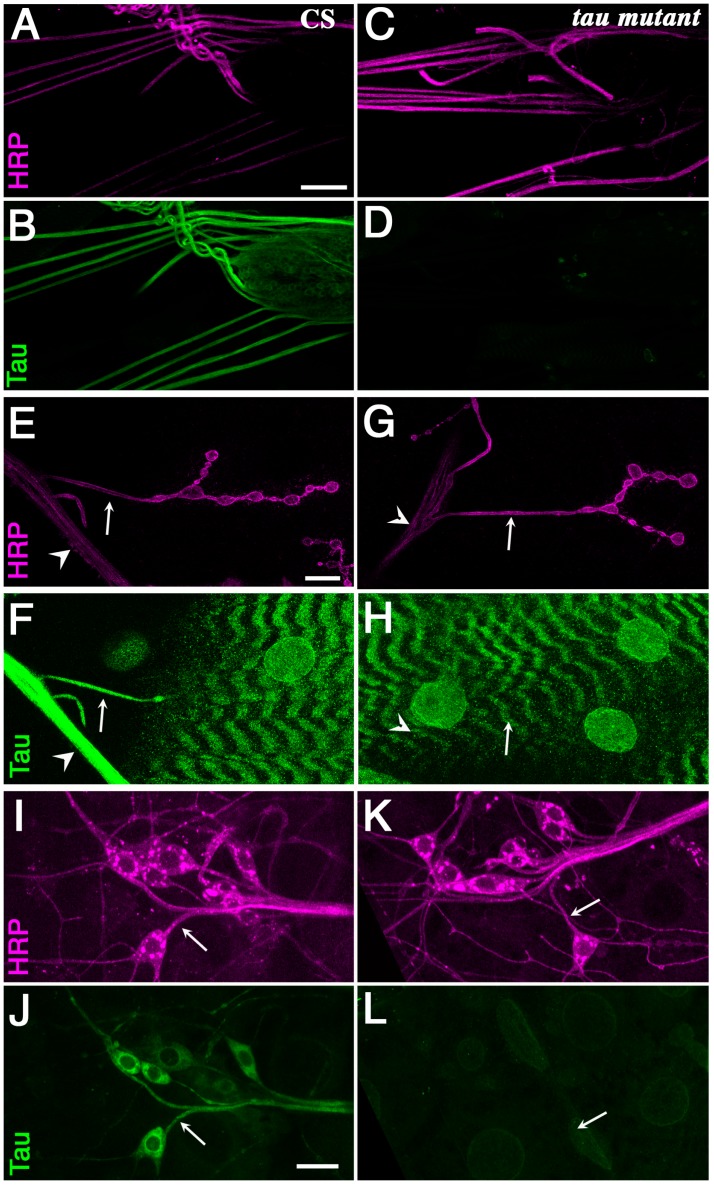
Tau is present in axons of motoneurons and sensory neurons. Anti Horse Radish Peroxidase (HRP) (magenta) and Tau (green) stainings of wild-type larvae (**A**,**B**,**E**,**F**,**I**,**J**) and *tau^ep3203^* larvae (**C**,**D**,**G**,**H**,**K**,**L**). The anti-HRP antibodies are known to specifically label neurons in insects. (**A**–**D**) larval nerves exiting the brain are immunoreactive with the anti-Tau antibody [34] in wild-type animal, but not in *tau* mutants. Scale bar is 50 μm. (**E**–**H**) Double HRP/Tau staining at the muscle 4 neuromuscular junction. There is some clear Tau staining in the axon (arrow) exiting the nerve (arrowhead) to innervate muscle 4 in wild-type larvae. There is no such staining in *tau* mutants. The staining observed in the underlying muscle is probably not specific since it does not disappear in *tau* mutants. Scale bar is 15 μm. (**I**–**L**) Double HRP/Tau staining in the dorsal cluster of sensory neurons. There is some clear Tau immunoreactivity in the axons (arrows) of the sensory neurons. There is no Tau immunoreactivity in *tau* mutants. Scale bar is 15 μm. CS: Canton S.

**Figure 2:**
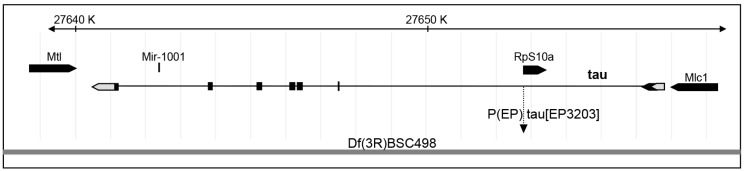
Genomic position of P{EP}tau[EP3203]. Relative position of the P element insertion P{EP}tau[EP3203] within *tau* gene and deficiency Df(3R)BSC498 on the right arm of the third chromosome. Left is proximal and the distances are indicated in kilobases. Exons are indicated by boxes, which are shaded in black when there is an open reading frame.

To investigate the role of endogenous *Drosophila* Tau in axons, especially its role in axonal transport, we focused on the segmental nerves of *Drosophila* larvae. These nerves provide an easy way to study axonal transport [38,39]. They contain axons of motor neurons as well as axons of the peripheral sensory neurons, which run in the opposite direction [40]. We used a previously published antibody against *Drosophila* Tau [34] to study Tau localization in these nerves (Figure 1). We tested the specificity of the stainings, by taking advantage of a known *tau* allele, the P element insertion *tau^ep3203^*, previously reported as a null or strong hypomorph allele because of the absence of wild-type protein, or any shorter protein, from embryonic extracts in Western blots [34] (Figure 2). We found clear immunostaining in the larval peripheral nerves of control larvae (Figure 1A). This staining was absent in mutant larvae (Figure 1B), indicating that it is specific for Tau. In order to know whether this Tau staining in segmental nerves corresponded to the presence of Tau in motor neuron axons or sensory neuron axons or both, we immunelabelled motor neuron axons at their exit of the nerve and muscle innervation endings. We found specific staining in the motor neuron axons (Figure 1C,D). We also immunolabelled sensory neurons, such as multidendritic sensory neurons, and saw again specific Tau staining in these axons (Figure 1E,F). Axons from other types of sensory neurons were also positive for Tau immunolabelling [41]. Hence, both motor and sensory neuron axons contain Tau in larval segmental nerves, suggesting that most axons present in the segmental nerves should be affected by *tau* depletion.

### 2.2. Tau Controls Microtubule Number in Larval Segmental Axons

**Figure 3:**
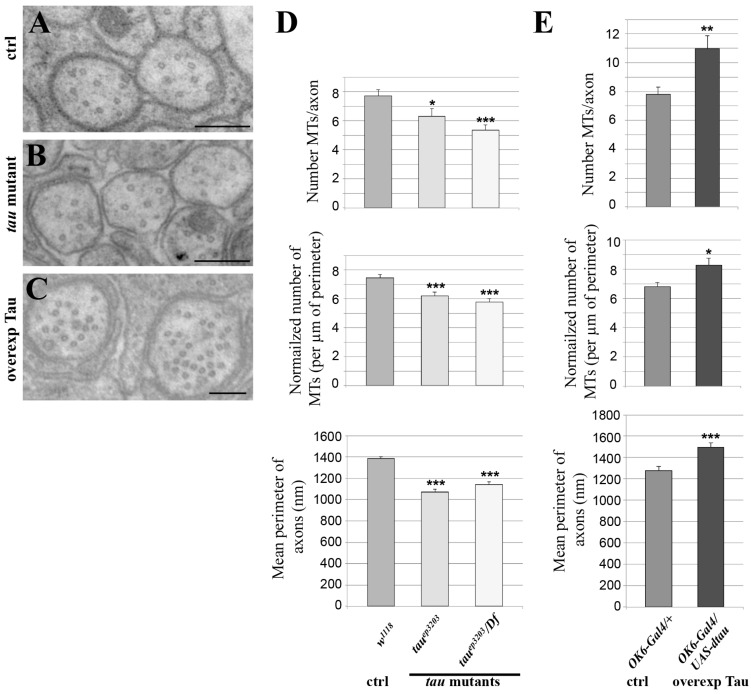
Effect of variation of Tau level on the microtubule cytoskeleton. Electron micrographs of segmental nerves axons in: (**A**) wild-type (*w^1118^*); (**B**) *tau* mutant larvae (*tau^ep3203^*); (**C**) *tau*-overexpressing larvae (in motoneurons: *OK6-Gal4*/UAS-*tau*). Scale bar is 200 nm (**A**–**C**). (**D**,**E**) Top histograms: mean number of MTs in segmental nerve axons. Middle histograms: normalized mean number of MTs in segmental nerve axons (number of MTs divided by perimeter of the measured axon). Bottom histograms: quantification of mean axonal size within segmental nerves, for different genotypes: (**D**) *tau* mutant and (**E**) *tau*-overexpressing larvae. Values are means +/− SEM. * *p* < 0.05, ** *p* < 0.01 and *** *p* < 0.001 (Student’s *t*-test).

To test whether endogenous *Drosophila* Tau is important for MT organization or density in axons of larval segmental nerves, we analysed two different genetic conditions for *tau* depletion: the P element insertion *tau^ep3203^*, which we found depleted for Tau in these tissues, either homozygous or trans-heterozygous with a deficiency encompassing the *tau* gene fully, Df(3R)BSC498 (Figure 2). We used transmission electron microscopy (TEM) on segmental nerves exiting the brain (Figure 3A–C) and when MTs could be detected unambiguously, we counted the number of those MTs per axon, The mean number of MTs per axon significantly decreased in the *tau* mutants compared to the control genotype (7.7 ± 0.4 MTs per axon in control *w^1118^* larvae (*n* = 117 axons) versus 6.3 ± 0.5 in *tau^ep3203^* mutants (*n* = 63 axons, *p* < 0.05) and 5.4 ± 0.3 MTs per axon (*n* = 112 axons, *p* < 0.01) in *tau^ep3203^/Df(3R)BSC498* mutants) (Figure 3D). Such a decrease was still observed when the number of MTs was normalized to the perimeter of the axon (7.5 ± 0.2 MTs per µm in control *w^1118^* larvae (*n* = 117 axons) versus 6.2 ± 0.3 in *tau^ep3203^* mutants (*n* = 63 axons, *p* < 0.001) and 5.8 ± 0.2 MTs per axon (*n* = 112 axons, *p* < 0.001) in *tau^ep3203^/Df(3R)BSC498* mutants) (Figure 3D). These data indicate that Tau is required for normal density of the MTs within axons. We also tested whether the loss of MTs in *tau* mutants was correlated to a reduced axon size by measuring the perimeter of all axons within each segmental nerve. Our data show that the mean perimeter of axons was significantly smaller in *tau* mutants compared to the control genotype (1384 ± 19 nm in control *w^1118^* larvae (*n* = 1280 axons, 6 larvae) versus 1069 ± 27 nm in *tau^ep3203^* mutants (*n* = 759 axons, 5 larvae, *p* < 0.001) and 1138 ± 27 nm in *tau^ep3203^/Df(3R)BSC498* mutants (*n* = 633 axons, 4 larvae, *p* < 0.001)) (Figure 3D).

We then tested whether an excess of *Drosophila* Tau was sufficient to increase MT number within axons. To that extent, we overexpressed Tau in the motor neuron axons, using the OK6-Gal4 driver, which leads to a 30-fold increase in the amount of *tau* transcripts, as estimated by qRT-PCR (Figure S1). In this condition, we found a significant increase in the number of axonal MTs as compared to wild-type nerves (7.8 ± 0.4 MTs per axon in control larvae (*OK6/+; n* = 69) versus 11.0 ± 1 in larvae overexpressing Tau (*OK6-Gal4/UAS-tau*; *p* < 0.01, *n* = 88)) (Figure 3C,D). Such an increase was still observed following normalization of the number of MTs to the axon perimeter (6.8 ± 0.3 MTs per µm in control (*OK6/+; n* = 69) versus 8.3 ± 0.5 in larvae overexpressing Tau (*OK6-Gal4/UAS-tau*; *p* < 0.05, *n* = 88) (Figure 3D), indicating that an excess of Tau leads to an increase in axon MT density. We also analysed whether, in the presence of an excess of Tau, the increase in MT number was correlated to axon enlargement. We found that the mean perimeter of axons within segmental nerves increased in condition of Tau overexpression as compared to the control condition (1277 ± 40 nm in control larvae (*OK6/+*, *n* = 330 axons, 4 larvae) versus 1494 ± 41 nm in larvae overexpressing Tau (*OK6-Gal4/UAS-tau*; *n* = 293 axons, 4 larvae, *p* < 0.001). Altogether, these in vivo data show that *Drosophila* Tau is required and sufficient to positively control MT number and MT density in axons as well as axon calibre.

### 2.3. Tau Depletion Does Not Affect Vesicle Density and Localization in Axons

Because axonal MTs enable anterograde and retrograde transport of organelles between the soma and the synaptic terminals, we undertook the analysis of axonal vesicular transport in *tau* mutants to test whether the observed decrease in MT density had an impact on this physiological function. We also analysed in parallel *tau*-overexpressing larvae.

**Figure 4:**
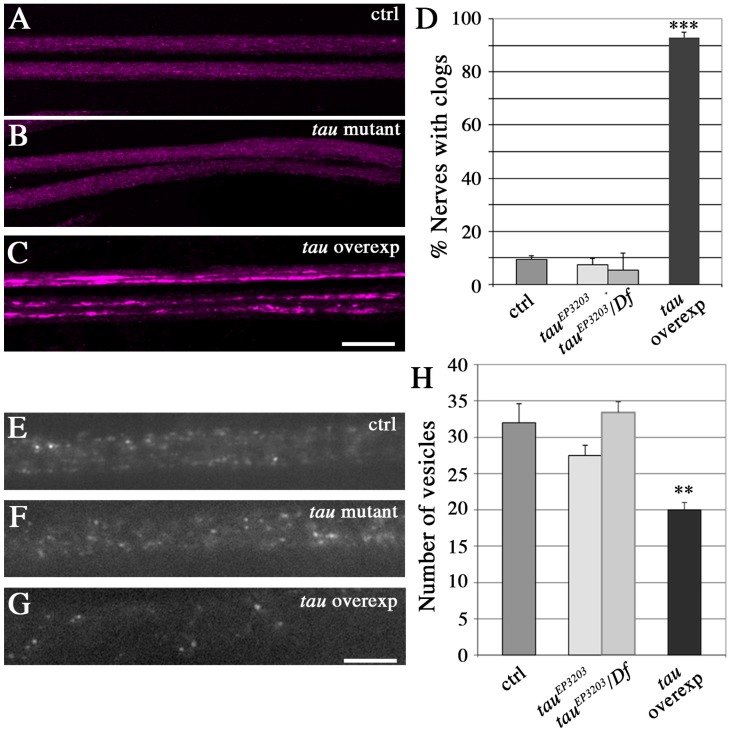
Effect of Tau depletion or excess on vesicle localization and density in axons. (**A**–**C**) Immunolabeling of vesicles with the anti-CSP (Cystein String Protein) antibody (Magenta) shows a massive presence of accumulations of vesicles, detected as intense fluorescent patches, in segmental nerves of *tau* overexpressing larvae, but no change of vesicle localization in *tau* mutants (*tau^ep3203^* mutant larvae). Scale bar is 25 μm. (**D**) Quantification of the relative number of nerves with vesicular accumulations (batches of vesicles) in different genotypes. The histogram shows a mean of 9.4 ± 1.5% of segmental nerves with accumulations of vesicles in control larvae (*n* = 20 larvae); 7.3 ± 2.4% of nerves with accumulations of vesicles (*n* = 21; non significant) in *tau^ep3203^* mutant larvae; 5.5 ± 6.2% of nerves with accumulations of vesicles (*n* = 9, ns) in *tau^ep3203^/Df(3R)BSC498* mutant larvae and 92.9 ± 2.5% of nerves with accumulations of vesicles (*n* = 12, *p* < 0.001) in *tau*-overexpressing larvae. Hence, only *tau*-overexpressing larvae display a significant increase in the number of accumulations of vesicles. Values are means +/− SEM. *** *p* < 0.001 (Student’s *t*-test), ns means non-significant. (**E**–**G**) In vivo visualization of vesicle density in segmental nerves by expressing the vesicular marker NPY-GFP in motoneurones shows a decreased density of vesicles only in *tau*-overexpressing larvae. In this last genotype, an image of a section of the segmental nerve without any batch of vesicles is shown (so that the higher intensity of these batches does not prevent from counting and visualizing of vesicles). Scale bar is 10 μm. (**F**) Quantification of the number of vesicles within a fixed nerve length (20 μm) in control flies, *tau* mutants, and *tau*-overexpressing flies. Only *tau*-overexpressing flies display a significant decrease in the number of “individual” vesicles (i.e., vesicles that are not stuck in batches). Only areas of nerves without accumulation of vesicles (the accumulation being upstream and downstream in the nerves) were selected for the counting of isolated vesicles in *tau*-overexpressing flies. Values are means +/− SEM. * *p* < 0.05 (Student’s *t*-test).

A phenotype commonly thought to result from disruption of vesicular transport is the accumulation of vesicles that are visible in immunocytochemistry as brighter and bigger fluorescent patches, within the segmental nerves. Such batches of vesicles are found in Kinesin mutants [42,43] and when Human or *Drosophila* Tau are overexpressed [44,45]. They are thought to correspond to vesicles that detach from microtubules and accumulate [46]. Here, we could not find such batches of vesicles in motor neuron axons of *tau* mutants, but we detected them in 90% of segmental nerves when *Drosophila* Tau was overexpressed (Figure 4A–D). This suggests that there was no major dissociation of vesicles from microtubules in *tau* mutants, and that Tau depletion does not lead to a strong phenotype, as it is the case for Tau overexpression. We also quantified the number of “individual” putatively mobile vesicles (i.e., vesicles that are not stuck within batches), within a given length of segmental nerve in live animals (20 μm). To that extent, vesicles were labelled by expressing a GFP-tagged neuropeptide (NPY-YFP) in motoneurons (using the OK6-Gal4 driver). There was no significant difference between control larvae and *tau* mutants, whereas Tau overexpression resulted in a significant decrease in the density of individual vesicles (Figure 4E–H): we counted a mean of 32 ± 2.6 vesicles in *w^1118^* larvae (*n* = 13 larvae), 27.5 ± 1.9 vesicles in *tau^ep3203^* mutants (*n* = 11; non significant), 33 ± 1.3 vesicles in *tau^ep3203^/Df(3R)BSC498* mutants (*n* = 8, ns) and 20 ± 1.4 vesicles in *tau* overexpressing larvae (*n* = 9, *p* < 0.01) (Figure 4D). This result shows that the amount of vesicles potentially moving along the MT tracks is unaffected in *tau* mutants, but is reduced in the presence of an excess of Tau. In conclusion, contrary to an excess of Tau, loss of Tau does not deplete potentially mobile vesicles within axons, nor does it lead to their accumulation into batches.

### 2.4. Tau Depletion and Tau Excess Differently Affect Vesicle Motion

We also analysed more comprehensively vesicle motion in *tau* loss of function or gain of function conditions, using the same transgenic flies expressing vesicular NPY-GFP. Motion of the fluorescent vesicles was tracked in live animals through the cuticle. To be able to distinguish between anterograde and retrograde movement of vesicles, we expressed this marker in motor neurons only (using the OK6-Gal4 driver). This technique was previously used to study the effects of Human Tau overexpression [47].

**Figure 5:**
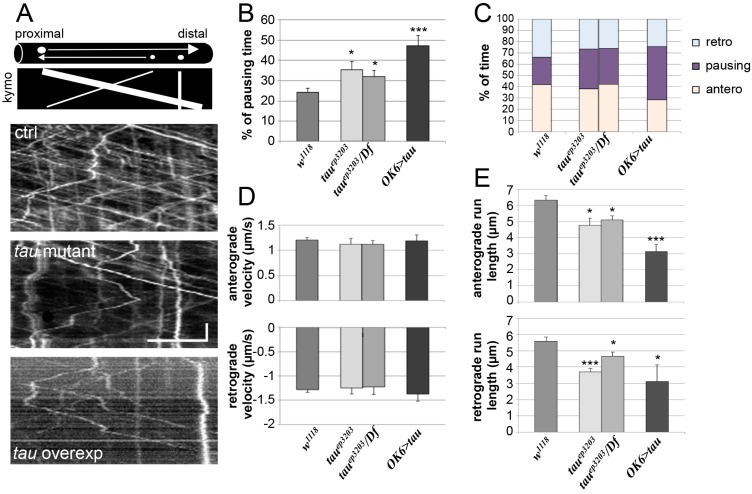
Vesicular axonal transport in *tau* mutants and *tau*-overexpressing larvae. (**A**) Examples of vesicular tracking analysis: for each genotype, a representative kymograph is shown. Kymographs allow the visualization of the movement of all vesicles within a selected axonal segment versus time. Diagram in (**A**) illustrates the trace of a vesicle moving anterogradely (e.g., with a large vesicle) or retrogradely (e.g., with a small vesicle). Immobile vesicles lead to vertical traces (e.g., with a medium size vesicle). The exact genotypes analysed were males *w/Y*; *OK6-Gal4/+*; *UAS-NPY-GFP/+* as the control, *w/Y*; *OK6-Gal4/+*; *UAS-NPY-GFP tau^ep3203^/tau^ep3203^* or *w/Y*; *OK-Gal46/+*; *UAS-NPY-GFP tau^ep3203^/Df(3R)BSC498* as the *tau* mutants and *w/Y*; *OK6-Gal4/UAS-dTau1*; *UAS-NPY-GFP/+*. Horizontal scale bar is 5 μm and vertical scale bar is 10 s. Histograms of the percentage of pausing (**B**), the percentage of time spent in anterograde or retrograde movement, or pausing (**C**), the instant anterograde (positive values) and retrograde (negative values) velocities for each genotype (**D**): *n* = 15 larvae for the control genotype; *n* = 11 and 7 larvae for *tau^ep3203^* mutants and *tau^ep3203^/Df(3R)BSC498* mutants respectively; *n* = 11 for *tau-*overexpressing larvae. For each larva, a number between 10 and 20 vesicles were tracked and their kinetic parameters quantified. (**E**) Histograms of the mean vesicle run length either for anterograde movement or retrograde movement: *n* = 6 larvae (83 vesicles) for the control genotype; *n* = 5 and 6 larvae (71 and 63 vesicles) for *tau^ep3203^* mutants and *tau^ep3203^/Df(3R)BSC498* mutants respectively; *n* = 5 larvae (74 vesicles) for *tau-*overexpressing larvae. Data are: mean +/− SEM. * *p* < 0.05, *** *p* < 0.001 (Student’s *t*-test).

We tracked the movement of several vesicles per studied nerve (Figure 5A) and measured different parameters of vesicle motion such as the percentage of time during which the vesicles do not move (pausing time), as well as the instant anterograde and retrograde velocities, the percentage of anterograde or retrograde movements, and the mean run length of vesicles. In control larvae, the mean pausing time of vesicles was 24.4 ± 1.9% (*n* = 15) (Figure 5B). In *tau* mutants, we observed a significant increase in pausing time of vesicles with 35.5 ± 4.0% pausing time in *tau^ep3203^* larvae (*n* = 11, *p* < 0.05) and 32.0 ± 3.0% pausing time in *tau^ep3203^/Df(3R)BSC498* mutants (*n* = 7, *p* < 0.05) (Figure 5B). There was no significant change in the relative amount of anterograde and retrograde transport (Figure 5C and Table S1). Also, there was no change in anterograde and retrograde instant velocities of vesicles in *tau* mutants as compared to control larvae (Figure 5D). When considering the mean run length of vesicles, we found a significant decrease for both anterograde and retrograde movements (Figure 5E): the mean anterograde and retrograde run lengths were 6.3 ± 0.3 and 5.6 ± 0.3 μm respectively in control larvae (*n* = 83 and 73, 6 larvae). They were 4.8 ± 0.5 μm (*p* < 0.05) and 3.7 ± 0.1 (*p* < 0.001) in *tau^ep3203^* larvae (*n* = 71 and 52, 6 larvae) and 5.1 ± 0.3 (*p* < 0.05) and 4.7 ± 0.3 (*p* < 0.05) in *tau^ep3203^/Df(3R)BSC498* mutants (*n* = 63 and 59, 5 larvae) (Figure 5E). In conclusion, even though Tau depletion does not induce axonal accumulation of vesicles, it actually does affect vesicular transport by increasing the pausing time of vesicles and decreasing the mean run length of both anterograde and retrograde movement. We compared these results to those obtained in the presence of an excess of Tau. In this condition, the mean pausing time of vesicles increased even more and reached 47.2 ± 5.3% (*n* = 11, *p* < 0.001; Figure 5B). Again, there was no significant change in the relative amount of anterograde and retrograde transport (Figure 5C and Table S1) as well as no significant change in instant retrograde and anterograde velocities of vesicles in *tau*-overexpressing larvae as compared to control larvae (Figure 5D). The mean run length of vesicles strongly decreased as compared to control larvae, reaching 3.1 ± 0.5 μm for anterograde movement (*p* < 0.001) and 3.1 ± 1.0 for retrograde movement (*p* < 0.05) (*n* = 74 and 67, 5 larvae) (Figure 5E).

In conclusion, both depletion and excess of Tau affect vesicular movement, by increasing the vesicles pausing time and decreasing mean run length, with more pronounced effect of Tau overexpression than depletion.

## 3. Discussion

This work aimed at studying the structural and functional consequences of loss of Tau, with respect to MTs and axonal transport, in *Drosophila* axons. Indeed, even though it is generally accepted that loss of Tau impacts the MT cytoskeleton and axonal transport, and people now focus on studying new MT-independent functions of Tau [48], there are actually few data underlying this assumption. In *tau* knock-out mice, only two studies reported a decrease in MT density: in cerebellar small calibre axons [24] and in the anterior commissural axons [48]. Similarly, there was no measurable defect in axonal transport of neurofilaments and SNAP25 in optic nerves of *tau* knock-out mice [26]. These data suggested that Tau loss may be compensated by other MAPs. Here, we used the model organism *Drosophila melanogaster*, in which there is less genetic redundancy. We used the *tau^ep3203^* mutant allele to analyse Tau depletion defects and found that there was no enhancement of the phenotypes observed in *tau^ep3203^/Df(3R)BSC498* mutants compared to homozygous *tau^ep3203^* mutants, suggesting that this *tau^ep3203^* allele behaves as a null allele with respect to the defects we studied. We observed a general decrease in MT density in the axons of the segmental nerves of *tau* mutant *Drosophila* larvae. Our data, added to those describing a decrease in MT density in axons of *tau* mutant *Drosophila* adult brain, using RNAi interference and a combination of deficiencies as *tau* mutant conditions [35], indicate that Tau is required for a normal density of MTs in this invertebrate organism. Remarkably, the observed decrease in MT number and MT density is about 20%, which is not such a strong defect considering that there is no possible functional redundancy within the MAP2/Tau family. This suggests that other microtubule-associated proteins—different from the MAP2/Tau family—are able to compensate for the depletion of Tau in these axons. This is in accordance with a recent report indicating that *tau* and the spectraplakin *shot* positively interact for the stability of MTs upon treatment with nocodazole [37]. Positive genetic interactions were also described between *tau* and the MAP1 *Drosophila* homologue, *futsch*, in controlling brain neurodegeneration, indicating that the corresponding proteins have at least partially overlapping functions [35]. Such a functional redundancy between Tau/MAP2 and MAP1 families was also described in mice, especially when considering microtubule organization in neurites [49,50].

When overexpressed in axons, we found that the *Drosophila* Tau induced a clear increase in MT number and MT density within axons, with the presence of bundles of MTs in which MTs are close to each other. This is reminiscent of MT bundles observed when Human Tau or MAP2 are overexpressed in cell lines [8,51] and shows the conservation of functional properties between *Drosophila* and Human Tau proteins.

Noticeably, the decrease in MT number and density was also accompanied by a decrease in axon calibre in *tau* mutants, and the increase in MT number and density was accompanied by an increase in axon calibre in *tau*-overexpressing larvae. To our knowledge, such a correlation between Tau-dependent number of MTs and axon calibre has not been previously described, either in *Drosophila* or in Vertebrates. It is possible that such an effect was detected in *Drosophila* because of the differences between factors controlling axon calibre in *Drosophila* and Vertebrates. Indeed, there is no *Drosophila* homologue of neurofilament proteins, known to be important for axon calibre in Vertebrates [52] and other large-size proteins like Ankyrin and Futsch may be involved in controlling axon calibre in *Drosophila* [53].

Because Tau depletion modified MT density in *Drosophila* axons, we wondered whether this could impact MT-dependent axonal transport, such as vesicular transport. We did not detect any accumulation of vesicles within batches in *tau* mutants as well as any decrease in the number of “individual” (potentially mobile) vesicles within nerves, i.e., vesicles that are not stuck within a batch and are thus potentially mobile. However, we did observe an increase in the pausing time of vesicles in *tau* mutants as well as a decrease in the run length of vesicles, whatever their direction. The pausing time defect could result from the observed decrease in MTs density and therefore the lower chance for a vesicle to find a nearby MT after reaching an MT end (hence the longer time spent pausing). One way to test this hypothesis would be to examine whether a decrease in microtubule density (independently of Tau) actually leads to an increase in vesicle pausing time. Also, the observed defect in vesicle movement could be linked to the known interaction between Tau and Dynactin, a member of the dynein complex involved in retrograde transport [54]. This could fit with the observed changes in mean run length of vesicles in *tau* mutants and *tau*-overexpressing larvae. Indeed, run length is known to depend on the number of motors bound to the cargo and their localization around the cargo [55,56,57]. Tau, by its binding to Dynactin or its association to vesicles [58,59], may have an impact on this. The fact that instant anterograde and retrograde velocities were unaffected by Tau depletion suggests that Tau does not play any limiting role in molecular motor (Kinesin or Dynein) kinetics. This is in accordance with several other reports showing that changes in Tau concentration along microtubules do not modify molecular motor kinetics [28,31,57,60,61].

In addition to increasing the number of axonal microtubules, Tau overexpression had strong effects on axonal transport, not only by increasing the pausing time of vesicles, similar to what was observed in condition of Tau depletion, but also by inducing accumulation of vesicles within batches in nerves and a subsequent decrease in the number of potentially mobile vesicles (i.e., individual vesicles). These phenotypes were also found in condition of Human Tau overexpression [39,47], indicating further that both Human and *Drosophila* Tau share conserved functions.

Because batches of vesicles possibly correspond to vesicles detaching from microtubules and accumulating in the axoplasm, our results suggest that an excess of Tau promotes vesicular detachment. This is in accordance with the in cellulo and in vitro experiments showing that MT-bound Tau increases molecular motor detachment (and hence, vesicle detachment), especially in the anterograde direction [28,29,60]. Indeed, when encountering patches of Tau-bound molecules on a microtubule track, Kinesin pauses and tends to detach, whereas Dynein tends to reverse direction [31]. Alternatively the N-terminal domain of Tau is of importance with respect to fast axonal transport [62,63]. Because this domain is involved in membrane association of Tau, especially in vesicles [58,59], it may be involved in accumulation of vesicles into batches observed in the presence of Tau overexpression. Hence, an excess of Tau impairs axonal transport in a different way compared to Tau depletion. Also, because of its effect on vesicle accumulation into batches, an excess of Tau is much more deleterious than a depletion of Tau for axonal transport.

The greater impact of Tau excess, compared to Tau depletion, on axonal transport may explain why there are several reports of neurodegenerative phenotype in the *Drosophila* eye or brain in condition of overexpression of *Drosophila* Tau [64,65] or Human Tau [66,67,68], but only one report of such a neurodegenerative phenotype in condition of loss of Tau function [35]. Interestingly this report shows a 5% retinal neurodegenerative phenotype in 28-day-old flies from the *tau* mutant allele *Df(3R)BSC499/Df(3R)MR22* (*trans*-heterozygous combination of two deficiencies overlapping within *tau* gene), in which a decrease in MT density was detected in adult brain axons. This suggests that the structural (MT density) and functional (axonal transport) defects observed in *tau* mutants may lead to a neurodegenerative phenotype in aged flies.

In conclusion, our data show that Tau depletion has consequences on microtubule density and axonal transport. They also show how neurons require Tau in order to get normal microtubule density and normal axonal transport but also may need to limit the amount of Tau bound to microtubules so that it does not sterically impair axonal transport. These two sides of Tau action on axonal transport may depend on the balance between MT-bound and MT-unbound Tau. This balance is physiologically regulated by post-translational modifications of Tau such as phosphorylation. How the high phosphorylation of Tau observed in neurodegenerative diseases plays a role on axonal transport, and to what extent this could be a causal effect in the neurodegenerative process remains to be further studied.

## 4. Materials and Methods

### 4.1. Fly Stocks

We used the mutant allele *tau^ep3203^* [34], as well as a deficiency encompassing the *tau* gene: *Df(3R)BSC498* (Bloomington Stock Center, Bloomington, IN, USA). Canton S or *w^1118^* flies were used as a wild-type control. For targeted expression of *Drosophila tau* gene, we employed the Gal4 activator strain OK6-Gal4 [69] and the UAS-dtau1 flies [65]. Crosses were performed at 25 °C. For vesicle tracking, we used the UAS-Neuropeptide Y-GFP (kind gift from Iain Robinson). This research was conducted under the agreement number 5722 (4 April 2011) from the Ministère de l’Enseignement Supérieur et de la Recherche, Direction Générale pour la Recherche et l’Innovation. 

### 4.2. Immunocytochemistry

Wandering third instar larvae were dissected in PBS 1x, EDTA 1 mM and then fixed for 20 min in 4% paraformaldehyde in PBS 1x. Immunostainings were performed in PBS 1x, 0.3%Triton X-100, 0.2% BSA. The following antibodies were used: polyclonal rabbit or goat anti-HRP (Sigma-Aldrich, St. Louis, MO, USA, 1:1000), polyclonal rabbit anti-Tau (kind gift from Daniel St Johnston), mouse monoclonal anti-DCSP-2 (DSHB, 1:150), polyclonal rabbit anti-synaptotagmin (kind gift from Hugo Bellen, 1:1000). The anti-DCSP-2(6D6) hybridoma developed by Simon Benzer was obtained from the Developmental Studies Hybridoma Bank, created by the NICHD of the NIH and maintained at The University of Iowa, Department of Biology, Iowa City, IA 52242. Fluorescent secondary antibodies were from Jackson ImmunoResearch, West Grove, PA, USA (donkey anti-rabbit Cy3, donkey anti-mouse Cy3, donkey anti-goat Cy5) or Molecular Probes (Invitrogen, donkey anti-rabbit Alexa 488 and donkey anti-mouse Alexa 488). Preparations were mounted in Vectashield media for observation. Confocal images were acquired from a Zeiss LSM 510 Meta confocal microscope (RIO imaging facility, Montpellier, France). Images were captured under oil with a 40× or 63× Plan-Apochromat objective, at excitation wavelengths of 488, 543, and 633 nm for GFP-tagged constructs or Alexa Fluor 488-, CY3- or CY5-conjugated antibodies, respectively. Band-pass and meta filters were applied as appropriate for respective fluorophores and images were captured from photomultiplier tube detectors (Carl Zeiss, Inc., Oberkochen, Germany).

### 4.3. Quantitative PCR Analysis

*Tau* transcript levels were measured using quantitative RT-PCR. cDNAs were generated from 1 μg total RNAs treated with DNase I by using random hexamers and Moloney murine leukaemia virus reverse transcriptase (LTI). Real-time PCR was done using Applied Biosystems (Courtaboeuf, France), SYBR Green PCR mix according to the manufacturer’s instructions. PCR was done as follows: 10 min at 95 °C followed by 40 cycles: 15 s at 95 °C, 60 s at 60 °C. Housekeeping genes used to normalize *tau* expression levels were RPL13, TBP and PGK. Sequences of the primers were: RPL13 5′-AGGAGGCGCAAGAACAAATC and 5′-CTTGCTGCGGTACTCCTTGAG (amplicon 72 nt), TBP 5′-CGTCGCTCCGCCAATTC and 5′-TTCTTCGCCTGCACTTCCA, PGK 5′-TCCTGAAGGTCCTCAACAACATG and 5′-TCCACCAGTTTCTCGACGATCT, Tau Exon 1 (S) 5′-TTGTCATTTACGCCGTTTCAAA and Tau Exon 1 (R) 5′-GGCTAGTCCTTTTCACTTGGAATAA.

### 4.4. Transmission Electron Microscopy

Third instar larvae were dissected in PBS 1x, EDTA 1 mM. Larval brains were then fixed overnight in 5% glutaraldehyde in 0.1 M phosphate buffer (NaH_2_PO4·2H_2_O and Na_2_HPO4·2H_2_O). They were then rinsed in phosphate buffer and post-fixed in a 1% osmic plus 0.8% potassium ferrocyanide for 2 h in the dark at room temperature. After two rinses in a phosphate buffer, the brains were dehydrated in a graded series of ethanol solutions (30–100%). The brains were embedded in EMBed 812 DER 736. Thin sections (85 nm; Leica-Reichert Ultracut E) were collected at different levels of each block. These sections were counterstained with uranyl acetate and lead citrate and observed using a Hitachi 7100 transmission electron microscope in the RIO imaging facility (C. Cazevieille, Montpellier, France).

### 4.5. Non-Invasive Tracking of Vesicles in Segmental Nerves

Larvae expressing NPY-GFP in their motor neurons were anaesthetised with ether over 2 min and 30 s. They were mounted in polymerizing 1% agarose between a slide and a coverslip, with their ventral face up. They were immediately placed under a 63× objective of an upright wide field fluorescent microscope to track vesicle motion within segmental nerves in segments A2–A3. A movie of 100 frames taken every 280 ms was recorded for one nerve of each larva. For each movie, we selected ~20 vesicles (on a fixed frame, i.e., not knowing if they were moving or not) and tracked them with the ImageJ plugin “manual vesicle tracking” developed by F. Cordelières (Bordeaux Imaging Center, Bordeaux, France). Percentage of pausing corresponds to the percentage of time during which vesicular instant velocity is inferior to 0.4 μm/s). Instant velocity is the velocity calculated between two consecutive time points: positive values correspond to anterograde movement, whereas negative values correspond to retrograde movement.

For each studied parameter, we analysed the distribution of the mean value obtained for each larva and could not reject the hypothesis that this distribution is normal. Hence, we compared the results obtained for each genotype using the Student’s *t*-test.

Quantification of run length was performed on kymographs obtained from each movie: a run was defined as a segment of trajectory with constant slope and separated from the next run by a change of slope. The Δx was measured for each run (and corresponds to the run length) of a vesicle trajectory.

## Supplementary Materials

The following are available online at http://www.mdpi.com/1422-0067/19/3/744/s1

## Abbreviations

HRP	Horse Radish Peroxidase
CSP	Cystein String Protein
MT	Microtubule
MAP	Microtubule-Associated Protein
