# Supplementary material for: Vesicular Axonal Transport is Modified In Vivo by Tau Deletion or Overexpression in Drosophila
Source: Int J Mol Sci. 2018 Mar 6;19(3):744. doi: 10.3390/ijms19030744 (PMC5877605; doi:10.3390/ijms19030744)
Supplement: Supplementary file 1 [file ijms-19-00744-s001.pdf]

Supplementary Materials

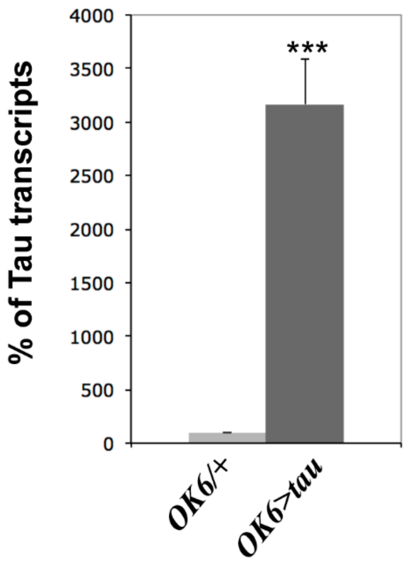

Supplementary Figure S1. quantitative RT-PCR on Tau overexpressing larvae. Histogram showing the relative percentage of *tau* transcripts (oligonucleotides within exon 1) in tau-overexpressing larvae (*OK6-Gal4/UAS-dTau1*) compared to the endogenous level of expression of *tau* in control larvae (*OK6-Gal4*/+), set at 100%. Results are mean +/− SEM of an experiment realized in triplicate. ***: $p < 0.001$ (Student's *t*-test).

	% pausing	% outward movement	% inward movement
ctrl	24.4 ± 1.9	41.7 ± 3.2	33.5 ± 2.9
ep(3)3203	35.5 ± 3.9*	38.0 ± 3.5 (p=0.45)	26.5 ± 2.2 (p=0.08)
ep(3)3203/Df(3R)BSC498	32.0 ± 3.0*	42.1 ± 4.4 (p=0.94)	25.8 ± 4.2 (p=0.15)
overexp Tau	47.2 ± 5.3***	28.5 ± 8.0 (p=0.1)	24.3 ± 5.2 (p=0.1)

Supplementary Table S1. Percentages of time spent in anterograde movement, pausing or retrograde movement. Data are: mean +/− SEM. *n* = 15 larvae for the control genotype; *n* = 11 and 7 larvae for tau^{ep3203} mutants and tau^{ep3203}/*Df(3R)BSC498* mutants respectively; *n* = 11 for *tau*-overexpressing larvae. *: $p < 0.05$, ***: $p < 0.001$ (Student's *t*-test).
